# Evolving perspectives of the role of novel agents in androgen-independent prostate cancer

**DOI:** 10.4103/0970-1591.42609

**Published:** 2008

**Authors:** Sujith Kalmadi, Derek Raghavan

**Affiliations:** Taussig Cancer Center, Cleveland Clinic, Cleveland, OH, USA

**Keywords:** Prostate neoplasm, chemotherapy, quality of life, bio-markers

## Abstract

Metastatic androgen-independent prostate cancer presents an intriguing clinical challenge, with a subtle interaction between hormone-responsive and refractory tumor cell elements. The treatment of advanced prostate carcinoma, which had remained stagnant for several decades following the understanding of the link between androgenic stimulation and carcinogenesis, has now started to make steady headway with chemotherapy and targeted approaches. Metastatic prostate cancer is almost always treated with initial androgen deprivation, in various forms. However, despite such treatment androgen-independent prostate cancer cells eventually emerge and progress to threaten life. The therapeutic objectives for treatment of metastatic prostate cancer are to maintain the quality of life and prolong survival. The out-dated nihilistic dogma of deferring chemotherapy until the most advanced stages in advanced prostate cancer is now falling by the wayside with the development of newer effective, tolerable agents.

## INTRODUCTION

Cytotoxic chemotherapy has evolved from the concepts of Lissauer and Ehrlich over the last century. The initial chemotherapy protocols devised by them were characterized by a lack of specificity and involved the fine balance of the toxicities experienced by the host and the tumor. This has been subsequently improved due to a better understanding of tumor biology and the biochemical basis of action of the chemotherapy regimens. Innovative modern techniques in the last score of years have provided further insight into the intracellular pathways that result in sensitivity and resistance of the neoplastic cells to drug treatment. This acquisition of new knowledge is occurring at a brisk pace resulting in a change in the understanding of the biology of the disease, a stage shift in clinical presentation and the development of highly accurate prognostic models.

In this review, we will discuss some of the important breakthroughs in the treatment of advanced prostate cancer [Table 1]. Metastatic androgen-independent prostate cancer presents an intriguing clinical challenge, with a subtle interaction between hormone-responsive and refractory tumor cell elements. The treatment of advanced prostate carcinoma, which had remained stagnant for several decades following the understanding of the link between androgenic stimulation and carcinogenesis by the pioneering work of Huggins and Hodges,[1] has now started to make steady headway with chemotherapy and targeted approaches.

**Table 1:** Therapeutic agents discussed in this article

Chemotherapeutic agents
Mitoxantrone Docetaxel Epothilones Oral platinums
Targeted agents
Vascular endothelial growth factor antagonist (Bevacizumab) Immunomodulatory agents (Thalidomide, Lenalidomide) Endothelin antagonists Vitamin D analogs Vaccines

## CHEMOTHERAPY FOR ANDROGEN-INDEPENDENT PROSTATE CANCER

Over the past decade, routine clinical use of prostate-specific antigen (PSA) has led to a stage migration in prostate cancer. This intense screening process has led to the detection of limited metastatic disease in the majority of patients. The analysis of outcome data from contemporary prospective clinical trials clearly reflects this lead-time bias, with an apparent improvement in survival in metastatic disease, due to earlier detection. This is an important concept when addressing whether recent progress in chemotherapy or other systemic treatment is real or an artifact of methodology.

Metastatic prostate cancer is almost always treated with initial androgen deprivation, in various forms. However, despite such treatment androgen-independent prostate cancer (AIPC) cells eventually emerge and progress to threaten life. Several mechanisms have been postulated for the acquisition of androgen independence.[2] The androgen receptor appears to be functioning and frequently overexpressed in AIPC, which may counteract the low levels of endogenous androgens. Activating mutations in the receptor gene, may alter ligand specificity, allowing other nonandrogenic steroids to activate the receptor. Cross talk between the androgen receptor and molecular pathways activated by growth factor receptors (i.e. HER2, insulin-like growth factor and epidermal growth factor receptor) can also contribute. Androgen ablation could also result in the transcription of death signaling genes without resulting apoptosis, due to downstream changes in the apoptotic pathways. Finally molecular changes within the prostate cancer cells, could convert a normally redundant signal transduction pathway into one which is uniquely required for proliferation.[3]

Chemotherapy trials in this setting are confounded by significant variables concerning the assessment of clinical response and benefit. The bulk of these patients will have metastatic disease confined to the bones. Reliability of radiologic examinations to distinguish a response in these osteoblastic bony lesions is limited. The absence of the soft tissue masses that characterize most solid tumors, which can usually be assessed with bidimensional measurements on radiological exams, severely hampers the use of commonly used “RECIST” criteria (developed by the U.S. National Cancer Institute) to assess response to treatment.[4] Clinical and laboratory parameters of performance status and pretreatment hemoglobin levels do correlate with eventual outcome.[5] Faced with these limitations, the National Prostate Cancer Project (NPCP) in the 1980s, was severely handicapped in providing accurate assessment of drug activity.

As a result of this, surrogate endpoints (PSA, improvement in pain and quality of life [QOL]) have been utilized in clinical trials to gauge clinical benefit.[6] Changes in serum PSA do appear to correlate with disease progression, treatment and survival. The PSA working group has recommended that objective responses in soft tissue mass, survival and a 50% decline in PSA, are all valid treatment endpoints for phase II clinical trials. However, the level of PSA decline, which best correlates with these endpoints, is highly controversial. Multivariate analysis of clinical trials, have demonstrated that a 50% decline has strongly correlated with survival.[7] The magnitude of serum PSA decline translating into clinical benefit is far from clear and in the recent SWOG 9916 (Southwest Oncology Group) trial, a sustained PSA decline of 30% was associated with a significant decrease in the risk of death.[8]

Also of importance is our inability to measure QOL improvements optimally. Most of the randomized trials cited below, have a surprising discordance between response, measured QOL, assessment of pain on structured quantification scales and long-term outcome.[9] Newer validated biomarkers are urgently needed to improve the efficiency of novel drug development. Another limitation of the utility of PSA measurement is that about 5-10% of patients will have a discordantly low level of PSA secondary to neuroendocrine differentiation of the tumor or may produce or release only negligible amounts of this protein. An important caveat in this subset of patients is that consideration should be given to the use of cytotoxic based initial treatment regimens, rather than conventional hormonal treatment of prostate cancer and it is also believed that cisplatin or carboplatin-based chemotherapy is more effective than some of the more traditional cytotoxics used for prostate adenocarcinoma [Table 2].[10]

**Table 2:** Summary of recent phase III trials of chemotherapy in androgen-independent prostate cancer

Chemotherapeutic regimen	Tannock *et al*.[6]		Petrylak *et al*.[8]	
		
	Docetaxel (3-weekly and prednisone)	Mitoxantrone and prednisone	Docetaxel and estramustine	Mitoxantrone and prednisone
Number of patients	335	337	338	336
Median age of the patients(age range)	68(42-92)	68(43-86)	70(47-88)	70(43-87)
Performance status	KPS < 70 (87%)	KPS < 70 (86%)	ECOG 0-1 (90%)	ECOG 0-1 (88%)
	KPS > 70 (13%)	KPS > 70 (14%)	ECOG 2-3 (10%)	ECOG 2-3 (12%)
Sites of disease:	Bone 90%	Bone 92%	Bone 84%	Bone 88%
	Soft tissue 22%	Soft tissue 22%	Soft tissue 26%	Soft tissue 26%
Median PSA (ng/ml)	114	123	84	90
Median survival	18.9 months	16.5 months	17.5 months	15.6 months
PSA response rates	45%	32%	50%	27%
Objective tumor responses (Radiologic)	12%	7%	17%	11%
Pain relief	35%	22%	Not reported. Not statistically different between the two arms	Not reported. Not statistically different between the two arms

## MITOXANTRONE

The therapeutic objectives for treatment of metastatic prostate cancer are to maintain the QOL and prolong survival. The out-dated nihilistic dogma of deferring chemotherapy until the most advanced stages in advanced prostate cancer is now falling by the wayside with the development of newer effective, tolerable agents. This long held dictum against the use of chemotherapy in prostate was first challenged by earlier trials with mitoxantrone, resulting in effective palliation of symptoms.[11] In this landmark phase III trial, the combination of mitoxantrone and prednisone, when compared with prednisone alone, demonstrated improvements in QOL with palliation of pain from bony metastases. The duration of palliative benefit was greater for patients receiving chemotherapy (43 vs. 18 weeks, *P* < 0.0001). Importantly however, this study allowed crossover, thus potentially vitiating any survival benefit. Even in the absence of a survival benefit, this led to the United States Food and Drug administration (FDA) to approve this agent for the treatment of symptomatic men with AIPC. Although the response rate of this combination has ranged from 33 to 48% in three large phase III randomized trials, a survival advantage has been lacking, although salvage chemotherapy may have negated the impact of initial mitoxantrone.[11–13]

Provocative data from our team have suggested that it may be possible to modulate the impact of mitoxantrone by the use of tesmilifene, a xenobiotic that alters the function of cytochrome P450 and which alters the function of multidrug resistance protein, thus altering the exposure of tumor cells to the active agent.[14] In a phase II trial, we demonstrated a surprising proportion of patients with very advanced, AIPC to be alive at 2 and 3 years, consequent upon an initial PSA response rate of more than 60%.[14]

## ESTRAMUSTINE

Estramustine phosphate is a synthetic nitrogen mustard derivative of estradiol which had demonstrated modest activity as a single agent in NPCP trials. It was initially hypothesized to act by targeting delivery of the mustard conjugate to malignant cells which overexpressed hormone receptors. Over time, insight into this agent's mechanism of action has shown that it acts via binding to microtubule-associated proteins, thus inducing microtubule destabilization. Based on preclinical modeling, it was combined with other antimicrotubule agents and topoisomerase poisons (i.e. vinca alkaloids, topoisomerase inhibitors and taxanes), to potentiate cytotoxicity. In the SWOG 9916 phase III trial, the combination of estramustine and docetaxel, resulted in a prolongation of survival, compared to mitoxantrone-based chemotherapy in AIPC.[15] However, the advantage was similar to what was achieved in the study known as TAX 327, with a less toxic regimen of docetaxel and prednisone.[6] With only a limited contribution to the docetaxel-based regimens, as well as the presence of thromboembolic events with estramustine, further clinical development of this agent has been substantially reduced.

## TAXANES

Recently two landmark randomized clinical trials, SWOG 9916 and TAX 327, with docetaxel, showed a modest improvement in survival in AIPC.[6,15] This demonstration of a survival advantage in advanced disease has also ushered in a new enthusiasm for the testing of docetaxel in all stages of prostate cancer.

SWOG 9916 compared the existing standard of care, mitoxantrone and prednisone, against the combination of docetaxel/estramustine based on the significant activity of this novel combination in prior phase II trials.[15] Patients were randomized to receive one of the following three-weekly regimens: (1) Mitoxantrone 12 mg/m^2^ on day 1 with prednisone 5 mg twice daily; or (2) docetaxel 60 mg/m^2^ on day 1, along with five consecutive days of estramustine 280 mg three times daily and 20 mg of dexamethasone daily for 3 days. Of the 674 eligible patients, the median age was 70 years, median PSA was 84 and bone pain was reported in two-thirds of the patients. Although the study failed to achieve the projected 33% improvement in survival, an intention to treat analysis revealed an improvement in median survival from 15.6 to 17.5 months (*P* = 0.02). The relative risk of death was decreased by 20% (Hazard ratio for death, 0.8; 95% CI 0.67-0.97). Median time to progression improved from 3.2 to 6.3 months (*P* < 0.001). While PSA response rates were statistically superior (27-50%), surprisingly this did not result in measured improvements in subjective pain relief rates. The docetaxel-estramustine treatment arm was more toxic, with eight treatment related deaths as compared to four in the mitoxantrone-prednisone arm. The incidence of grades 3 or 4 gastrointestinal (20 *vs*. 5%, *P* < 0.001), hematologic (neutropenic fever, 5 *vs*. 2%; *P* = 0.01), cardiovascular (15 *vs*. 7%, *P* = 0.001) and neurologic events (7 *vs*. 2%; *P* = 0.001) was greater in the docetaxel-estramustine arm as compared to the mitoxantrone-prednisone arm.

The TAX 327 phase III trial, compared dose equivalent docetaxel given either on a weekly basis or every 3 weeks, against mitoxantrone.[6] The three chemotherapy regimens: (1) Mitoxantrone 12 mg/m^2^, (2) docetaxel 75 mg/m^2^ every 3 weeks and (3) docetaxel 30 mg/m^2^ every week for 5 of 6 weeks. Patients on all arms received prednisone 5 mg twice daily. Of the 1006 patients enrolled in the trial, the median age was 68 years, median PSA ranged from 108 to 123 ng/ml and approximately 45% of the patients had pain. Based on an intention to treat analysis, the median durations of survival were 18.9 months in the three-weekly docetaxel arm, 17.4 months in the weekly docetaxel arm and 16.5 months in the mitoxantrone arm. Three-weekly, but not weekly docetaxel had a statistically significant survival benefit with a hazard ratio for death of 0.76 (0.62-0.94, 95% CI). This was very similar to the hazard ratio of 0.80 obtained in the SWOG trial. Analysis of secondary endpoints revealed reduced pain (35 *vs*. 22%), PSA response (45 *vs*. 32%) and QOL improvement. Common adverse events in the three-weekly docetaxel regimen which occurred more frequently included fatigue, diarrhea, neutropenia and neuropathy [Table 2].

The complementary results from these two trials reported concurrently in the *New England Journal of Medicine*, definitively established that docetaxel-based chemotherapy prolongs median survival and that it achieved a 20% mortality reduction over the study period. While the improvements were modest, they were equivalent to early studies in breast cancer that opened the way to the use of cytotoxic chemotherapy as adjuvants to definitive local therapy. These data establish a backbone on which to develop improved regimens for metastatic or high risk, locally advanced prostate cancer.

## NOVEL STRATEGIES

In an effort to build on the demonstrated success of docetaxel, investigators have explored a broad range of traditional cytotoxic agents and novel-targeted molecules which may be additive or synergistic. With the recent discovery of novel pathways involved in prostate cancer progression, progress has been made in the understanding of the biology of AIPC. This has signaled the dawn of an era where novel combinations may further slow progression and convert aggressive prostate cancer to a more benign phenotype, that will allow patients to die with, rather than of prostate cancer.

Among the cytotoxic agents, the ones which have garnered more enthusiasm are the epothxilones and the oral platinums. Epothilones are structurally different from the taxanes, but act similarly by stabilizing the polymerized microtubule. There are several epothilone analogues in clinical trials in AIPC. Ixabepilone (BMS-247550), an epothilone B analog, is the farthest along in clinical trials and has exhibited PSA response rates of 39-48% in phase II trials in chemotherapy naïve patients.[16] Satraplatin is a novel oral platinum with modest activity in AIPC. Preclinical data suggest activity in taxane-resistant prostate cancer cell lines. In an aborted phase III trial, comparing satraplatin plus prednisone *vs*. prednisone, there was an apparent improvement in progression free survival from 2.5 to 5.2 months (*P* = 0.023).[17] An international, multicenter trial, termed the SPARC5 trial, designed to test this improvement in survival, will lend further insight about the activity of this agent when the data are mature.

Differentiation therapy with agents that reverse the dedifferentiation that accompanies the malignant phenotype, provide an alternative to conventional combination chemotherapy, because of the favorable side effect profile. High-dose calcitriol (Vitamin D3) in combination with docetaxel has generated much interest based on favorable preclinical data and phase II studies. In addition to its differentiating properties in prostate cancer cell lines, calcitriol has been shown to inhibit the growth, reduce invasion and angiogenesis, stimulate apoptosis and is synergistic with chemotherapy.[18] In an interim report of the phase III ASCENT (AIPC study of enhancing taxotere) trial, while the primary endpoint of PSA response was not met, there was an intriguing improvement in overall survival, although it must be emphasized that further follow-up will be critical to the interpretation of these data.[19]

Angiogenesis is a promising target in many malignancies, based on the early work performed by Folkman.[20] Antiangiogenesis therapies are hypothesized to be effective in preventing tumor-associated neoangiogenesis and normalization of existing microvasculature thereby improving drug delivery to the tumor.[21] To date, the most successful use of antiangiogenic agents has been seen in combination with conventional chemotherapy in colon and lung cancer. Bevacizumab is a monoclonal antibody directed against the vascular endothelial growth factor (VEGF) which has shown synergism with cytotoxic chemotherapy, resulting in a survival advantage in lung and colon cancer. VEGF is mitogenic for prostate cancer cells and is androgen regulated. Higher levels of VEGF have correlated with a poorer outcome. Encouraging phase II activity of this agent with docetaxel, has led to a phase III trial of the Cancer and Acute Leukemia Group B (CALGB), which is currently underway. Thalidomide is a molecule with a multifaceted mechanism of action including antiangiogenesis, immunomodulation and inhibition of platelet derived growth factor. Possible synergy between docetaxel and antiangiogenic therapy has been demonstrated in a randomized phase II trial with thalidomide where there was an apparent improvement in PSA response rate from 38 to 53%, although a structured randomized trial will be required to demonstrate true utility.[22] The toxicities in this trial, largely vascular in nature, make the issue of determining the benefit of adding thalidomide to docetaxel extremely challenging. Newer analogs of thalidomide (i.e. Lenalidomide and CC-4047), with a safer toxicity profile are being investigated in this setting.[23]

Endothelins (ET-1, ET-2 and ET-3) are a group of peptides that are produced in a wide variety of tissues, where they serve as integral modulators of vasomotor tone, cell proliferation and signal transduction. Prostate cancer is characterized by the loss of endothelin receptors and increased endothelin levels, which in turn leads to inhibition of apoptosis.[24] ET-1 is critically important in the pathogenesis of osteoblastic bony metastasis, due to activating mitogenic changes. A large phase III trial with single-agent atrasentan (Abbott Laboratories), an oral ETA receptor antagonist, was stopped early due to lack of efficacy in AIPC.[25] However in a preliminary report of a metaanalysis, AIPC patients treated with atrasentan were 14% less likely to experience disease progression (HR = 0.86, 95% CI 0.75-0.99), had an 18% less likelihood of experiencing bony pain (HR = 0.82, 95% CI 0.69-0.98) and had a 22% less chance of experiencing PSA progression (HR = 0.78, 95% CI 0.69-0.98).[26] This has given rise to a randomized trial under way through the SWOG 0421, which compared docetaxel plus atrasentan *vs*. docetaxel alone for AIPC.

Other promising novel pathways which are being explored in early trials include epidermal growth factor receptor inhibitors, multiple tyrosine kinase inhibitors, farnesyl transferase inhibitors, rapamycin kinase inhibitors, proteasome inhibitors and histone deacetylase inhibitors, but no definitive data are yet available.

## IMMUNOTHERAPEUTIC APPROACHES

Immunotherapy for prostate cancer is an active field of investigation with two major approaches: active and passive immunotherapy. Active immunotherapy involves the delivery of a molecule to elicit an immune response (e.g. vaccines), while passive immunization involves the delivery of a molecule with intrinsic immunologic activity (e.g. antibodies).[27]

Vaccines against prostate cancer can be separated into autologous (the individual patient's own tumor cells are used) and allogeneic (tumor cells from established tumor cell lines). APC 8015 (Provenge) is a vaccine that consists of autologous dendritic cells that have been pulsed *ex vivo* with a prostatic acid phosphatase-granulocyte macrophage-colony stimulating factor (GM-CSF) fusion protein. In a preliminary report of a phase III trial in asymptomatic AIPC, comparing Provenge or placebo, while the primary endpoint of progression free survival was not met, there was an improvement in overall survival (25.9 *vs*. 21.4 months; *P* = 0.02).[28] To rule out the potential bias from a statistical artifact, these results are being verified in a multicenter trial. Prostate cancer vaccine (GVAX) is an allogeneic vaccination strategy in which prostate cancer cells are genetically engineered to secrete high levels of GM-CSF, then mixed with autologous patient mononuclear cells *ex vivo* and reinfused. Preliminary data suggest that this approach may delay time to progression in AIPC, although the overall impact on survival is not yet clear.[29]

## NEUROENDOCRINE CARCINOMA OF THE PROSTATE

Neuroendocrine transformation of prostate cancer is reported in a small subset of men with advanced AIPC. These tumors tend to have several unique characteristics, including the absence of androgen receptors and PSA production and the presence of a variety of growth factor receptors (i.e. bombesin, somatostatin, chromogranin-A, serotonin and Parathyroid hormone (PTH)).[30] This leads to a clinical presentation quite different from AIPC, with more frequent visceral and soft tissue metastases, osteolytic bone metastases, brain metastases, hypercalcemia and a rapid clinical deterioration. Histologically, they will have a small-cell differentiation or a poorly differentiated carcinoma with neuroendocrine markers. These tumors should be treated similar to other high-grade neuroendocrine carcinomas (e.g. small-cell carcinoma of the lung) with a combination of platinum and etoposide or equivalent regimens.[31,32]

## FUTURE DIRECTIONS IN AIPC

Improvements in survival with currently available chemotherapeutic approaches have been modest. The dawn of an exciting era of drug development is upon us, representing a treatment paradigm shift. An expanding portfolio of newer targeted agents, have lent themselves to testing in this setting. Enhancements in understanding of the molecular pathways over the last few decades, have resulted in targeted agents, which can be combined with classic cytotoxic agents. Recognizing the multifactorial nature of drug resistance and the inherent survival mechanisms has made us realize that collateral and downstream pathways can circumvent targeting single mechanisms of drug resistance. Bypassing these adaptive cellular changes would be better served with a quiver full of arrows. While none of the newer treatment modalities has yet been shown to be more effective than standard treatment, the potential armamentarium is steadily growing. Combining cytotoxic chemotherapy with the newer targeted agents, raise the appealing plausibility of parallel or possibly complementary, therapeutic effects. Efficient trial design, appropriate selection of correlative markers, greater cooperation between urologists and medical oncologists and close toxicity monitoring will propel this field further and improve our management of this disease.
